# Cylindrospermopsin-Microcystin-LR Combinations May Induce Genotoxic and Histopathological Damage in Rats

**DOI:** 10.3390/toxins12060348

**Published:** 2020-05-26

**Authors:** Leticia Díez-Quijada, Concepción Medrano-Padial, María Llana-Ruiz-Cabello, Giorgiana M. Cătunescu, Rosario Moyano, Maria A. Risalde, Ana M. Cameán, Ángeles Jos

**Affiliations:** 1 Area of Toxicology, Faculty of Pharmacy, University of Sevilla, Profesor García González n2, 41012 Sevilla, Spain; ldiezquijada@us.es (L.D.-Q.); cmpadial@us.es (C.M.-P.); mllana@us.es (M.L.-R.-C.); angelesjos@us.es (Á.J.); 2 University of Agricultural Sciences and Veterinary Medicine Cluj-Napoca, Calea Mănăștur 3-5, 400372 Cluj-Napoca, Romania; giorgiana.catunescu@usamvcluj.ro; 3 Department of Pharmacology, Toxicology and Legal and Forensic Medicine, Faculty of Veterinary Medicine, University of Córdoba, Campus de Rabanales, 14014 Córdoba, Spain; ft1mosam@uco.es; 4 Animal Pathology Department. Faculty of Veterinary Medicine, University of Córdoba, Campus Universitario de Rabanales s/n, 14014 Cordoba, Spain; maria.risalde@uco.es; 5 Instituto Maimonides de Investigación Biomédica de Córdoba (IMIBIC)-Hospital Universitario Reina Sofía de Córdoba-Universidad de Córdoba, Avenida Menendez Pidal s/n, 14006 Cordoba, Spain

**Keywords:** in vivo, genotoxicity, cylindrospermopsin, microcystin-LR, micronucleus, comet assay, enzyme-modified comet assay, rats

## Abstract

Cylindrospermopsin (CYN) and microcystins (MC) are cyanotoxins that can occur simultaneously in contaminated water and food. CYN/MC-LR mixtures previously investigated in vitro showed an induction of micronucleus (MN) formation only in the presence of the metabolic fraction S9. When this is the case, the European Food Safety Authority recommends a follow up to in vivo testing. Thus, rats were orally exposed to 7.5 + 75, 23.7 + 237, and 75 + 750 μg CYN/MC-LR/kg body weight (b.w.). The MN test in bone marrow was performed, and the standard and modified comet assays were carried out to measure DNA strand breaks or oxidative DNA damage in stomach, liver, and blood cells. The results revealed an increase in MN formation in bone marrow, at all the assayed doses. However, no DNA strand breaks nor oxidative DNA damage were induced, as shown in the comet assays. The histopathological study indicated alterations only in the highest dose group. Liver was the target organ showing fatty degeneration and necrotic hepatocytes in centrilobular areas, as well as a light mononuclear inflammatory periportal infiltrate. Additionally, the stomach had flaking epithelium and mild necrosis of epithelial cells. Therefore, the combined exposure to cyanotoxins may induce genotoxic and histopathological damage in vivo.

## 1. Introduction

Climate and nutrient changes are contributing to global eutrophication and global expansion of harmful algal blooms [1], including the proliferation of cyanobacterial blooms [2]. Cyanobacteria are producers of a broad group of secondary metabolites called cyanotoxins [3]. The main human exposure route to cyanotoxins is oral intake, primarily from drinking water. However, the consumption of contaminated food and dietary supplements with cyanotoxins cannot be disregarded, although some of them (such as cylindrospermopsin) have not been found in commercially-available blue-green algal supplements until recently [2,4,5]. The most studied cyanotoxins are microcystins (MCs) and cylindrospermopsin (CYN) as a consequence of their toxicity and wide distribution. The severity of these intoxications depends on several factors, such as their chemical structure, mechanisms of action, and concentrations of cyanotoxins involved [2].

MCs are cyclic heptapeptides that are mainly hepatotoxic [6], including as primary target organs the liver and kidney, as well as several secondary targets such as testes, ovaries, heart, lung, and the CNS; to date 246 MC congeners have been identified [7]. Microcystin-LR (MC-LR) is the most studied MC variant, because of its higher toxicity and wider distribution than other MC congeners [8,9]. Hepatotoxicity of MC-LR is mediated by the presence of organic anion transport polypeptides (OATPs) which are expressed mostly in the liver and are responsible for its uptake into the hepatocytes [10]. MC-LR is a specific inhibitor of protein serine/threonine phosphatases 1 and 2A (PP1, PP2A) [11]. It induces a hyperphosphorylation of cytoskeletal proteins affecting cell morphology and cellular adhesion, leading to necrosis [12,13]. Oxidative stress is another mechanism involved in MC toxicity [14,15,16,17]. Therefore, MC-LR could have carcinogenic or genotoxic properties because of its inhibitory action on PP1 and PP2A, acting as a liver tumor-promoter [18,19,20]. In fact, MC-LR was classified only as a possible human carcinogen (Group 2B) by the International Agency of Research on Cancer (IARC) [21], mainly because of the lack of sufficient evidence for its direct carcinogenicity in both humans and experimental animals [2].

The genotoxicity of pure MC-LR cyanotoxin has been widely studied and reviewed in the scientific literature [20,22]. Globally, contradictory results were reported following in vitro and in vivo experimental systems, and the mechanisms involved are not yet fully understood. Pioneering studies indicated that MC-LR damaged the DNA in the liver of male and female Swiss albino mice dependent upon the assayed dose and on the duration of exposure after intraperitoneal injection (i.p.) [23]. This effect was observed after just a single administration of a LD_50_ dose of MC-LR [6,24]. Likewise, DNA damage was observed in the blood cells of Swiss albino mice after oral exposure to a single dose of MC-LR. Higher damage was found in different organs after i.p. injection [25]. Moreover, MC-LR induced a rapid increase of the amount of DNA in the tail of the comets and increased micronucleus (MN) frequencies in male mice injected i.p. [26] and in Balb/c mice exposed repeatedly to MC-LR for 30 d [27]. By contrast, other studies reported that MC-LR did not induce DNA damage in vivo. Thus, no DNA damage was observed in rat liver after a single intravenous administration (i.v.) of MC-LR [28], and negative results were observed in the MN assay in male transgenic mice after intragastric administration [29], and in male CBA mice after i.p. injection of MC-LR [19].

CYN is a stable tricyclic alkaloid consisting of a guanidine moiety combined with a hydroxymethyluracil [30], whose occurrence in aquatic systems is increasing. This cyanotoxin can be produced by different cyanobacterial genera such as *Aphanizomenon, Cylindrospermopsis, Lyngbya, Oscilliatoria, Raphidiopsis and Umezaki*, but *Cylindrospermopsis raciborskii* is its main producer [31,32]. CYN has cytotoxic activity and its effects on the liver and other organs have been proven [2,33]. The main mechanism of CYN toxicity is the inhibition of both protein and glutathione synthesis [34,35,36], although oxidative stress is also involved [37]. Several studies have shown that cytochrome P450 enzymes are necessary in the metabolic activation of CYN, and consequently in its toxicity [38,39]. 

At present, the mechanisms of pro-genotoxicity and potential carcinogenic activity of CYN (not yet classified by the IARC) are still not completely described and, thus, further investigations are needed. To this end, several in vivo studies in rodents were performed to clarify the genotoxicity of CYN. Thus, CYN induced DNA strand breaks (sb) in the liver of mice after i.p. administration [40]. Similarly, DNA damage was observed in the colon of mice after i.p. injection of the toxin, and both in the colon and bone marrow after oral (gavage) administration [41]. Dordevic et al. [42] demonstrated DNA damage (comet assay) in the liver of rats exposed i.p. to CYN and to an extract of *Cylindrospermopsis raciborskii*. Recently, Diez-Quijada et al. [43] exposed rats to pure CYN (7.5–75.0 µg/kg b.w) and performed a battery of assays consisting of MN in bone marrow, as well as the standard and modified comet assays in stomach, liver and blood. The DNA seemed to be damaged only in the bone marrow of rats regardless of concentration. By contrast, neither DNA strand breaks nor oxidative DNA damage was observed in the comet assays in any of the investigated tissues.

Both MC-LR and CYN can be found at the same time in the environment and their simultaneous presence was previously described [44,45,46]. Thus, the European Food Safety Authority (EFSA) documented and stated the importance of studying the effects of their combined exposure [47]. A few studies dealt with the toxicological profile of CYN and MC-LR combinations. Thus, the in vitro assessments of the potential interactions of CYN and MC-LR are very scarce [48,49,50,51], and only two focus on their genotoxicity. Hercog et al. [48] described an induction of DNA sb by MN and comet assays at 24 h after the treatment of HepG2 cells with CYN/MC-LR combinations, but to a lesser extent for only CYN. The genomic instability detected by the cytokinesis block micronucleus assay was, however, comparable to the individual CYN. Recently, Diez-Quijada et al. [49] applied a battery of in vitro tests in several cell lines, including bacterial systems, and they described genotoxic effects only in the MN test, when the metabolic fraction S9 was used. To the best of our knowledge, no in vivo studies have been yet performed to assess the genotoxicity of CYN/MC-LR mixtures and they are necessary to elucidate the contradictory results obtained in vitro.

Thus, according to the recommendations of the EFSA [52], the purpose of this research was to investigate, for the first time, the potential in vivo genotoxicity of CYN/MC-LR combinations in rats, as an experimental model, after oral administration (gavage) of relevant environmental concentrations. A combined MN—standard and modified comet assay was applied. The enzyme-modified comet assay was performed with Endonuclease-III (Endo-III) and Formamidopyrimidine glycosilase (Fpg) enzymes. Bone marrow was the selected tissue for the MN test, Organisation for Economic Co-operation and Development (OECD )474 [53], and stomach, liver OECD 489 [54] and blood cells for the standard and enzyme-modified comet assays. Additionally, the potential histopathological alterations were assessed in stomach and liver.

## 2. Results

### 2.1. Micronucleus Assay

This assay was conducted following the recommendations of OECD guideline 474 [53], and it is especially relevant for assessing genotoxicity because, although they may vary among species, factors of in vivo metabolism, pharmacokinetics, and DNA repair processes are active and contribute to the responses. Its purpose is to identify cytogenetic damage which results in the formation of micronuclei (MN) containing lagging chromosome fragments or whole chromosomes. As positive control, ethylmethanesulfonate (EMS) was chosen according to this guideline. Results are measured as the polychromatic erythrocytes (PCE) out of total erythrocytes (normochromatic erythrocytes (NCE) + (PCE)), and the PCE/NCE ratios, which were calculated by counting 500 erythrocytes per animal. An increase in the frequency (%) of micro-nucleated polychromatic erythrocytes (%MN-PCEs) in treated animals is an indication of induced chromosome damage.

The results obtained for the MN test in rats exposed to CYN/MC-LR mixtures are shown in Table 1, with individual data shown in the Supplementary Materials (Table S1). Significant differences versus the negative and solvent control groups were found in the PCE/total erythrocytes and PCE/NCE ratios in male and female rats treated with the highest assessed dose (75 + 750 µg/kg b.w. CYN/MC-LR; **p < 0.01). Treatment with the positive control, ethylmethanesulfonate (EMS), produced similar significant decreases in the PCE/total erythrocytes and PCE/NCE ratios. Furthermore, significant increases in the percentage of MN in immature erythrocytes were observed in all treated groups of both sexes, when compared with the negative and solvent control groups.

### 2.2. Standard and Enzyme-Modified Comet Assay

The standard comet assay can detect single and double stranded breaks (SBs), resulting, for example, from direct interactions with DNA, alkali labile sites or as a consequence of transient DNA strand breaks resulting from DNA excision repair [54]. Moreover, the enzyme-modified comet assay allowed for detection of oxidative DNA damage using the enzymes Endonuclease III (EndoIII) and Formamidopyrimidine DNA glycosylase (Fpg), which detect oxidized pyrimidines and purines, respectively. The results are expressed as the DNA content in the tail (% of DNA in the tail), which is the intensity of the comet tail relative to the total intensity. As positive control, ethylmethanesulfonate (EMS) was chosen.

**Figure 1:**
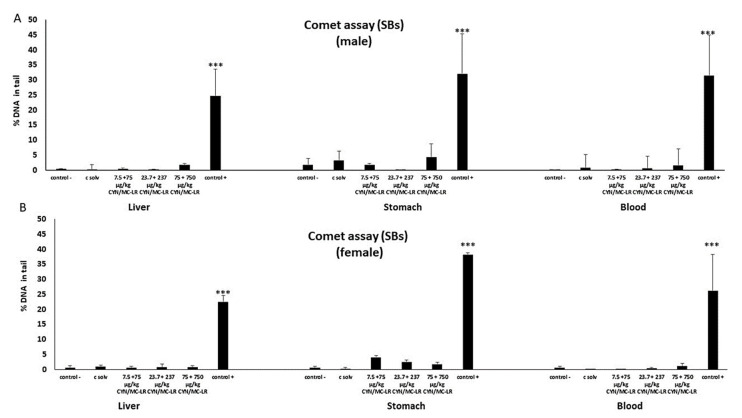
The level of DNA damage in cells isolated from liver, stomach, and blood of male (**A**) and female (**B**) rats exposed to cylindrospermopsin/microcystins (CYN/MC-LR) mixtures as the formation of strand breaks (SBs) detected by the standard comet assay. The levels of DNA strand breaks are expressed as % DNA in the tail of the comets. All values are represented as mean ± SD. Significantly different from control (*** *p* < 0.001).

**Figure 2:**
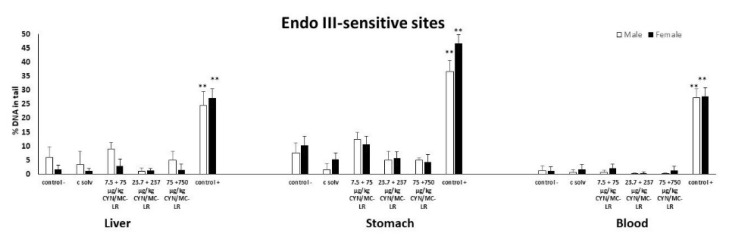
The level of DNA damage in cells isolated from liver, stomach and blood of male and female rats exposed to CYN/MC-LR combinations as the formation of oxidative DNA damage in the form of Endo-III sensitive sites. The levels of oxidized pyrimidines are expressed as % DNA in the tail of the comets. All values are represented as mean ± SD. Significantly different from control (** *p* < 0.01).

**Figure 3:**
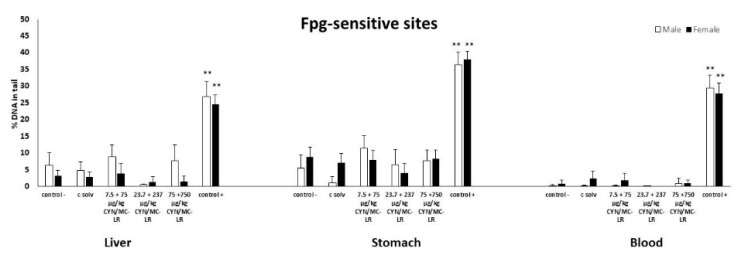
The level of DNA damage in cells isolated from liver, stomach, and blood of male and female rats exposed to CYN/MC-LR mixtures as the formation of oxidative DNA damage in the form of Formamidopyrimidine glycosilase (Fpg)-sensitives sites. The levels of oxidized purines are expressed as % DNA in the tail of the comets. All values are represented as mean ± SD. Significantly different from control (** *p* < 0.01).

No DNA strand breaks were induced in the standard comet assay at any assessed dose in liver, stomach, and blood cells, with the exception of rats treated with the positive control (EMS) (Figure 1A,B). Furthermore, no increases were observed in the % of DNA in the tail of the comets in liver, stomach and blood cells of both sexes at any exposure assayed after post-treatment with Endo-III (Figure 2). Similarly, no differences were found in the tissues from the treated and control groups after Fpg post-exposure (Figure 3). Significant DNA damage was observed in the studied tissues obtained in all the experiments in the positive control group treated with 200 mg/kg b.w. of EMS, (Figure 1, Figure 2 and Figure 3). Individual data of alkaline and enzyme-modified comet assays are shown in the Supplementary Materials (Table S1). Summary statistics for the treatment groups (male + female) of both assays are also included in the Supplementary Materials (Table S2). 

### 2.3. Clinical and Histopathological Analysis

No clinical signs of toxicity were detected during the experiment at any assayed dose of CYN/MC-LR combinations. No macroscopic changes were observed after necropsy in the gastric mucosa or liver samples of treated groups of both sexes, whereas the stomach had an increased size in the positive control groups. The relative weight (RW) of liver (excised wet liver weight/animal weight) and stomach collected from the treated animals with cyanotoxins was similar to that of the control groups (see Tables S3 and S4 in Supplementary Materials).

**Figure 4:**
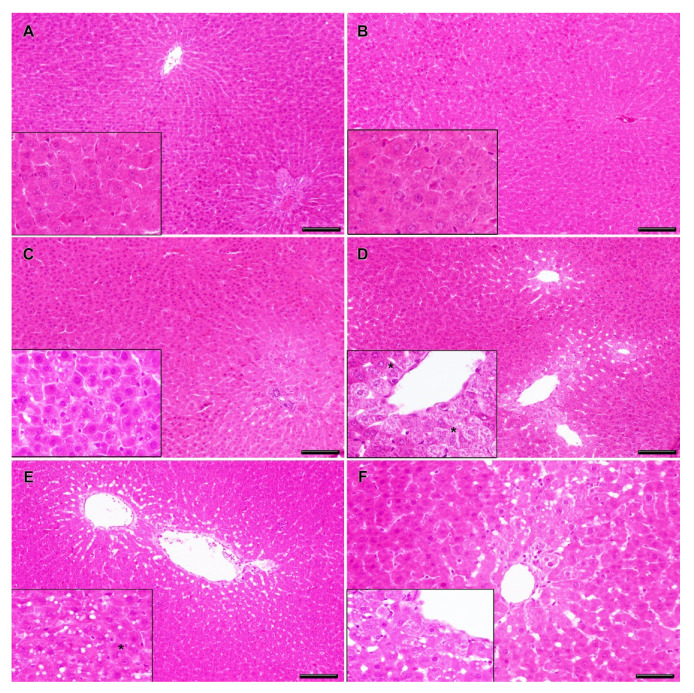
Representative histopathological changes in the liver of rats exposed to CYN/MC-LR. Normal hepatic parenchyma is observed in negative (**A**) and solvent (**B**) control groups. Details of normal hepatocytes are observed in the insets (**A**,**B**). Rats exposed to 7.5 + 75 µg/kg b.w. CYN/MC-LR showed a diffuse distribution of mild degenerate hepatocytes with micro-vesicular lipid vacuolation (**C**). There are details of intracellular accumulation of small, round and clear vacuoles in hepatocytes (**C**, inset). Rats exposed to 23.7 + 237 µg/kg b.w. CYN/MC-LR presented mild degenerate and necrotic hepatocytes in centrilobular areas (**D**). There are details of degenerate hepatocytes, some of them multinucleated (*) (**D**, inset). Rats exposed to 75 + 750 µg/kg b.w. CYN/MC-LR showed moderate degenerate and necrotic hepatocytes in centrilobular areas with macro-vesicular lipid vacuolation (**E**). There are details of intracellular accumulation of large, round, and clear vacuoles inside hepatocytes, some of them multinucleated (*) (**E**, inset). The positive control group showed hepatocyte degeneration with lipid accumulation and centrilobular necrosis (**F**). There are details of the degeneration of hepatocytes with macro-vesicular lipid vacuolation (**F**, inset). Hematoxylin and eosin staining; bars = 100 μm (**A**–**E**) and 50 μm (**F**).

**Figure 5:**
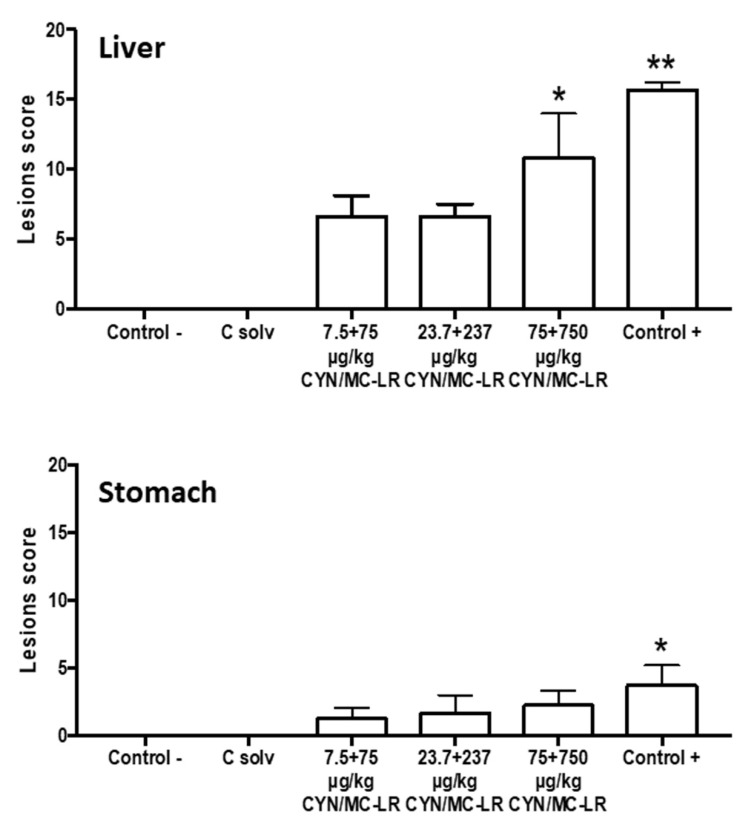
Mean ± standard error (SE) of the histopathological lesion score from liver and stomach of rats exposed to CYN/MC-LR. Significantly different from control (* *p* < 0.05; ** *p* < 0.01).

The liver and stomach from the negative and solvent control groups showed normal histology without pathological lesions (Figure 4A,B). Degenerate hepatocytes in centrilobular areas together with small, round, and clear vacuoles (microvesicular vacuolation) inside hepatocytes with a diffuse distribution were observed in the liver from rats exposed to medium-lower doses (23.7 + 237 and 7.5 + 75 µg/kg b.w. CYN/MC-LR), (Figure 4C,D); however, they did not reach statistical significance with regard to the negative and solvent controls when quantified (Figure 5).

Similar, but more severe liver lesions than in the lower doses were observed in the highest exposed group (75 + 750 µg/kg b.w. CYN/MC-LR). In this case, degenerate and necrotic hepatocytes in centrilobular areas were observed, as well as ample intracellular lipid accumulation in hepatocytes in the form of round and clear vacuoles (macrovesicular vacuolation). These had a multifocal distribution together with mild presence of multinuclear hepatocytes (Figure 4E). This group showed significant liver damage compared to negative and solvent control groups (Figure 5). 

The liver pathology of the positive controls was characterized by severe centrilobular necrosis, hepatocyte degeneration with lipid accumulation, and mild mononuclear inflammatory periportal infiltrates (Figure 4F). This group showed significant differences in the lesion scores compared to negative and solvent control groups (Figure 5).

**Figure 6:**
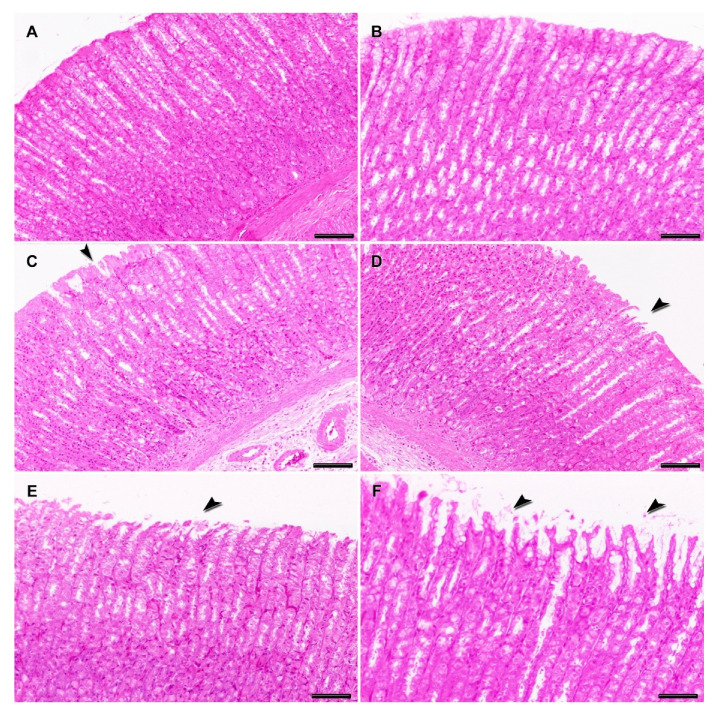
Representative histopathological changes in the stomach of rats exposed to CYN/MC-LR. Normal gastric mucosa is observed in negative (**A**) and solvent (**B**) control groups. Rats exposed to 7.5 + 75 and 23.7 + 237 µg/kg b.w. CYN/MC-LR showed minimal flaking gastric epithelium (arrows) (**C**,**D**, respectively). Rats exposed to 75 + 750 µg/kg b.w. CYN/MC-LR showed a mild flaking gastric epithelium with occasional necrosis of epithelial cells and a mild increase of the secretory epithelium (arrow) (**E**). Positive control group presented a moderate flaking gastric epithelium with mild necrosis of epithelial cells and hyperplasia of the gastric glands (arrows) (**F**). Hematoxylin and eosin staining; bars = 100 μm (**A**,**C**,**D**), 75 μm (**B**,**E**) and 50 μm (**F**). The lesions were independently examined by two experienced assessors: a veterinary pathologist and an investigator (M.A.R. and R.M.) in a single-blinded approach. These findings were evaluated in all the fields of one slide with a section of 1 × 1 cm from each organ studied in all the animals.

On the other hand, the main damage in the stomach included flaking epithelium and minimal-mild necrosis of epithelial cells with different severity, depending on the groups. These lesions were moderate in the positive controls accompanied by hyperplasia of the gastric glands, while being mild in the highest exposed group, minimal in the medium-lower doses, and totally absent in negative and solvent controls (Figure 6A–F). Statistical differences were observed only between positive control and the rest of the studied groups (Figure 5).

## 3. Discussion

Although the simultaneous occurrence of cyanotoxins such as MCs and CYN is being reported more and more frequently [44,55], toxicological studies focusing on their potential interaction are very scarce [50,56]. Particularly, the genotoxicity of mixtures is of great interest for humans exposed to contaminated waters and food with cyanotoxins, due to the possible carcinogenic effects of MC-LR and pro-genotoxic activity of CYN. Moreover, as EFSA recommends [47], further studies are needed to characterize the hazard for a more realistic and reliable risk assessment. 

Only two in vitro studies evaluated the genotoxic effects of CYN/MC-LR mixtures. The first was carried out on the HepG2 cell line exposed to MC-LR (1 µg/mL), CYN (0.01, 0.05, 0.1, and 0.5 µg/mL) and their mixtures by comet and MN assay [48]. The authors indicated that CYN might have a higher genotoxic potential than MC-LR and the genotoxic potential of CYN/MC-LR combination was similar to CYN alone. More recently, the mutagenicity and genotoxicity of 1:10 CYN/MC-LR mixtures were assayed through a complete battery of in vitro tests including the MN test on L5178Y Tk^±^ cells and the standard and enzyme-modified comet assays in Caco-2 cells [49]. Genotoxicity was observed only for the combination in the MN test with S9 metabolic fraction, in agreement with the previous reports for only CYN [57]. Together, both in vitro studies suggested the predominance of the pro-genotoxic activity of CYN in the combinations, hence the necessity to evaluate in vivo the genotoxicity of CYN/MC-LR mixtures by application of international guidelines such as the OECD guidelines. 

This is the first in vivo study which reported the genotoxicity of CYN/MC-LR in rats orally exposed (by gavage). It combines and relates the results of the two types of comet assays (standard and enzyme-modified) on cells isolated from stomach, liver, and blood, with the MN test in bone marrow cells. The assays were performed according to the OECD 489 and 474 guidelines, respectively [53,54] with some alterations, as described by Diez-Quijada et al. [43]. The combined comet-MN assay [58] reduces the use of animals according to the 3Rs principles (Replace, Reduce, and Refine), and it increases the sensitivity and specificity of the assays, decreasing the number of false negative results [59]. Moreover, in this case, the use of DNA repair enzymes (Endo III and Fpg) was also included, increasing the sensitivity of the in vivo comet assay [60,61].

In this study, the combined MN-comet assay was performed in both sexes, because differences in toxicity linked to gender have previously been described. This is in agreement with the OECD 489 [54] that encourages the use of both sexes when relevant differences between males and females are reported (e.g. differences in systemic toxicity, metabolism, bioavailability, etc. including those in a range-finding study). In this sense, although no sex-related differences were observed in rats after MC-LR i.p. exposure [62], male mice were more sensitive than females to CYN administered i.p. [63]. CYN exposure doses (75, 23.7, and 7.5 µg/kg b.w.) were selected according to a previous in vivo study performed with the individual toxin [43]. The doses of MC-LR were 10 times higher than CYN because of its proportionally higher abundance in the environment [64]. Overall in this study, using rats as experimental model, the CYN/MC-LR combinations increased the % MN-PCE in bone marrow cells, although it induced no DNA strand breaks nor any oxidative DNA damage in any of the investigated tissues. These results are in accordance with the only two previous genotoxicity in vivo assay carried out in rats with pure individual toxins: CYN alone under the same combined assays [43] and MC-LR administered i.v. through the comet assay [28]. Generally, the results of this study show no synergism or antagonism between CYN and MC-LR in the assayed combinations and concentrations in rats, because the effects are similar to the in vivo exposure to CYN alone [43]. Moreover, these findings confirm the in vitro experiments performed with the CYN/MC-LR mixture through a battery of tests, in which MN and comet assays were included [49]. 

In the present study, the significant decreases in the PCE/total erythrocytes and PCE/NCE ratios in the highest dose groups (75 + 750 µg/kg b.w. CYN/MC-LR) in comparison to the controls were consistent with results observed for CYN tested individually at the same doses (7.5–75.0 µg/kg b.w.) [43]. Previously, in the case of MC-LR no in vivo effects were reported for polychromatic erythrocytes (PCE) from peripheral blood of male mice after i.p. administration of the toxin (0–55 µg/kg b.w.) nor induction of MN [19].

Moreover, the induction of MN by the CYN/MC-LR mixture in rats contrasts with the contradictory results obtained in previous assays performed in mice as experimental model. Thus, negative results were reported in the colonic cells of mice orally exposed (1–4 mg/kg b.w.) and in bone marrow after i.v. administration [41]. In contrast, positive results were found in the blood cells of mice exposed i.p. to 37.5 µg/kg b.w. MC-LR, with significant increases in the frequency of MN at 48–72 h after treatment [26]. Additionally, only a rapid and temporary two-fold increase had been detected in the amounts of DNA in the tail of the comets after 30 min of MC-LR exposure was detected in the comet assay. The authors suggested that although MC-LR induced DNA damage, the leukocytes might repair the lesions, prior to the genotoxicity assessment by the comet assay. They concluded that the MN assay could be more sensitive than the comet assay to evaluate the genotoxicity of MC-LR. This hypothesis could also explain the results obtained in the present work. Other studies showed a significant dose-dependent increase in MN frequency of bone marrow cells of Balb/C mice i.p. injected with MC-LR (0.5–8 µg/kg b.w.) every 48 h for 30 d [27]. In contrast, an in vivo negative MN induction was found in CBA mice exposed i.p. to pure MC-LR [19]. Moreover, MC-LR did not show mutagenicity (in lung and liver), and it did not induce gene mutation nor MN in peripheral blood cells of transgenic λ/lacZ mice at 48 h after exposure to 1 mg/kg b.w. MC-LR [29]. The authors suggested that the tumor-promoting effect of MC-LR is independent of its interactions with DNA.

Overall, the in vivo induction of MN by the CYN/MC-LR combinations observed in this study confirms the genotoxicity demonstrated previously in vitro, and corroborates the reported in vitro pro-genotoxic activity of CYN [39,57,65,66,67], and to a lesser degree of MC-LR [26,68]. Moreover, previous in vivo studies resulted in similar effects for CYN whereas the diversity of results obtained for MC-LR could be attributed to different animal species, exposure routes, and doses used. Our results were obtained on rats, the experimental model recommended by the OECD guidelines on genotoxicity [53,54] and toxicity studies; and they clarify previous contradictory findings reported in mice.

The application of the alkaline comet assay in stomach, liver, and blood cells is a good complement to the bone-marrow and peripheral blood MN test [58] because this combination allows assessment of the DNA damage in various potential target tissues (site of contact, metabolism and peripheral distribution) and it can detect multi-endpoint genotoxic effects [59]. In the present study, the absence of effects is in consensus with a previous in vivo combined study carried out with CYN alone in male rats under similar experimental conditions [43]. 

In contrast, to the best of our knowledge, the few in vivo comet assays available revealed positive results [40,41,42]. However, it must be emphasized that CYN was administered i.p., and this could influence the higher intensity of the results obtained: induction of DNA sb in the liver of mice [40], in the colon of mice [41], and in the liver of rats [42]. Nonetheless, the oral route is the most representative for human exposure to cyanotoxins [2,5,33]. In this sense, only Bazin et al. [41] found DNA damage in colon and bone marrow samples from mice exposed by gavage to CYN in a range of 1–4 mg/kg b.w., doses much higher than in this study. 

Previous in vivo studies reported contradictory results for individual and pure MC-LR from the alkaline comet assay. Thus, there were DNA lesions in the blood cells (strand breaks, labile sites, etc.) of mice at 3 h after oral exposure to MC-LR (4 mg/kg b.w.). However, DNA lesions were observed mostly in the liver after i.p. administration, although they were also induced in the kidney, intestines, and colon [25]. The authors concluded that the DNA damage induced in the organs was probably due to oxidation, and it could be attributed to the more sensitive i.p. route of administration. Similarly, the DNA breaks were reported in blood cells of mice exposed i.p. to MC-LR (37.5 µg /kg b.w. MC-LR) [26]. In contrast, MC-LR did not induce DNA damage in rat hepatocytes at 2–4 h or 12–16 h after exposure to a single sublethal doses (ranging between 12.5–50 µg/kg b.w.) administered i.v. [28]. The authors suggested that the administration route influences the results from the in vivo comet assay and changes the MC-LR kinetic parameters. Further in vivo combined MN-comet assay for MC-LR should be carried out in rats, in order to know the genotoxic profile of this toxin.

Moreover, the oxidative stress could be one of the genotoxic mechanisms of CYN and MC cyanotoxins [20]. Thus, the modified-comet assay was also performed in this study. No oxidative damage was observed in the pyrimidine and purine bases of DNA, in agreement with the negative results found in the in vivo experiment performed at the same doses with CYN alone in male rats [43]. Moreover, it corroborates the results reported in vitro on Caco2 cells exposed to CYN/MC-LR mixtures [49], and the scarce results obtained from in vitro enzyme-modified comet assay for CYN in HepG2 cells [69] and in Caco-2 cells [57]. This suggests that oxidative stress could have a minor role in the CYN mediated genotoxicity [22,69].

In contrast to CYN, MC-LR tested in vitro showed increased DNA strand breaks by oxidation of pyrimidine and purine bases in HepG2 cells [16,70], providing evidence that the toxin induced strand breaks from excision of oxidative DNA adducts. Thus, reactive oxygen species (ROS) are involved in this type of DNA damage. The same authors confirmed that MC-LR displayed oxidation of purine bases in human peripheral blood lymphocytes when DNA was digested with purified Fpg. Moreover, oxidative stress-responsive genes were up-regulated at the molecular level after 24 h, supporting the hypothesis that MC-LR is an indirect genotoxic agent, acting via induction of oxidative stress [71].

The discrepancy between the negative results of the present study on the DNA oxidative damage induced by the mixtures and previous reports on individual MC-LR suggests that the genotoxic activity of CYN rather than MC-LR is responsible of the effects of CYN/MC-LR mixtures in rats. In fact, Hercog et al. [48] who studied CYN/MC-LR combinations in HepG2 cells concluded that MC-LR did not seem to deregulate the investigated genes, and they suggested the higher pro-genotoxic potential of CYN. However, MC-LR was classified in the group 2B by the IARC [21] due to its tumor promotion mechanism. Thus, caution is required when assessing its toxicity when in the mixture. 

The liver was the main target organ of the CYN/MC-LR mixtures as showed by the histopathologic evaluation. It was also the most severely affected organ in MC-exposed fish [72], rats [73,74], and mice [75,76]. The major findings of oral toxicity in the present experiment were fatty generation and necrotic hepatocytes in centrilobular areas, as well as a light mononuclear inflammatory periportal infiltrate, specially noted in the highest doses of toxins (75 + 750 μg/kg b.w. CYN/MC-LR). This degeneration and the necrotic processes of hepatocytes were similar to those reported in experimental administrations of MC-LR in mice [75,77,78] and rats [74]. Thus, our results indicate that MC-LR is undeniably incorporated into the liver, resulting in the characteristic hepatotoxicity and confirming the sensitivity of this organ to the oral administration of MC-LR. Further studies with additional inflammatory and hepatotoxic markers (gene and/or protein expression) should be performed to support the histopathological analysis. These in vivo results are in accord with other in vivo and in vitro studies where MC-LR induced liver injury through the production of ROS among other mechanisms [74,79,80]. Hepatocyte injury was also observed after CYN exposure in rats [42,43,81] and mice [41,63,82,83], with its severity increasing with the dose [37,63,82,83,84]. On the other hand, a variable number of multinucleated hepatocytes was observed in the high and medium-dosed groups (23.7 + 237 or 75 + 750 μg/kg b.w. CYN/MC-LR) implying the presence of liver injuries. However, this histological change was not observed in the low-dosed group. An increase in the mitotic frequency was also described in rats exposed only to the highest CYN dose (75 μg/kg b.w.) [43]. Nevertheless, other liver injuries associated with MC-LR, such as fibrosis, were not observed upon histological examination. MC-LR is known to induce liver fibrosis in pre-clinical models and in people after acute exposure to MC [14,85,86]. However, these findings are normally associated with a more severe liver damage and advanced disease [74].

In the present study, mild histopathological findings were recorded in the stomach of all exposed groups. In contrast, significantly altered gastric mucus secretions were found previously after oral administration of MC-LR in mice [87] or pure CYN in rats [43]. These lesions were associated with an irritant effect of the toxins since the gastric mucus is the first line of defense against luminal irritants [43,88]. However, the discrepancies could be associated with the age of the animals [87] or the time of exposure, which was longer in the study of Diez-Quijada et al. [43].

## 4. Conclusions

The study performed provided evidence of an enhancement of MN formation in the bone marrow of rats subsequent to an oral exposure to CYN/MC-LR combinations in the range 7.5 + 75 to 75 + 750 µg/kg b.w. However, no genotoxic damage was observed in other organs such as liver, stomach, and blood as evaluated by the standard and enzyme-modified comet assay. Therefore, the combined exposure to cyanotoxins may induce genotoxic damage in vivo, although there is no evidence of synergistic or additive effects due to their combination. Histopathological lesions were observed mainly in the liver, in agreement with the well-known hepatotoxicity of these cyanotoxins. These results support the demand for further confirmatory studies needed for a thorough risk assessment of cyanotoxins and their mixtures, and consequently for possible risk management measures limiting human exposure if required.

## 5. Materials and Methods 

### 5.1. Chemicals and Reagents

Microcystin-LR standard (99% purity) and Cylindrospermopsin standard (95% purity) were acquired from Alexis Corporation (Lausen, Switzerland). All assay chemicals were obtained from C-Viral S.L. (Seville, Spain), Gibco (Biomol, Seville, Spain), Moltox (Trinova, Biochem, Germany) and Sigma-Aldrich (Madrid, Spain).

### 5.2. Animal Housing and Feeding Conditions

The Ethics Committee on Animal Experimentation of the University of Sevilla approved this in vivo experiment (09/03/2016/027). In addition, all animals received care following the Directive 2010/63/UE for the protection of animals used for scientific purposes.

Male and female Wistar rats, strain RjHan:WI (type outbred rats), between seven and eight weeks old were purchased from Animal Production and Experimentation Service (SEPA, University of Cádiz). Animals were weighed on the arrival day (weight variation did not exceed ± 20%) and housed into polycarbonate cages with stainless steel covers. Afterwards, the animals were fed during one week before the experiments with standard laboratory diet (Harlan, 2014; Harlan Laboratories, Barcelona, Spain) and water *ad libitum*. During this time, animals were acclimatized to the environmental conditions with a 12 h dark/light cycle, temperature 23 ± 1 °C, relative humidity (55 ± 10)%, and free from any type of chemical contamination.

### 5.3. Experimental Design and Treatment

The treatment doses of CYN were selected based on our previous study of genotoxicity following oral administration of 75 µg CYN /kg b.w. for three days [43]. The chosen concentrations of MC-LR were 10 times higher than CYN because MC are proportionally more abundant in nature than CYN [2,89]. Even though occurrence data show that MC-RR and other minority MCs are distributed worldwide becoming sometimes predominant, the congener MC-LR is widely distributed and the main focus of toxicological studies [8,64]. The ratio CYN/MC concentrations could oscillate between countries and continents, climatic conditions, or composition of the cyanobacteria communities [44,45], and in this study the ratio 1:10 CYN/MC-LR was chosen to compare or confirm previous toxicity studies in which combinations of CYN/MC-LR were assayed [49,50,51]. Furthermore, the International Conference on Harmonisation (ICH) S2 guidelines [90] and the OECD 474 [53] and OECD 489 [54] guidelines for the MN and Comet assay, respectively, recommend for the combined MN-comet assay the use of the highest dose and two additional lower doses [58] appropriately separated by less than √10 to prove dose-related responses [54]. Thus, increasing concentrations of CYN/MC-LR mixtures were selected: 7.5 + 75, 23.7 + 237, and 75 + 750 μg/kg b.w. CYN/MC-LR, respectively, according to Diez-Quijada et al. [43]. These doses are not only experimentally relevant (according to the OECD guidelines), but also environmentally significant, especially the lower ones. Thus, lower doses of 23.7 or 7.5 μg CYN/kg, b.w. are very relevant, being the equivalent to an exposure of 3.4 μg or 1.0 μg CYN/rat/day, respectively.

In this study, 28 male and 28 female rats were randomly divided into six groups, three controls and three treatment groups: the negative control group (C-) (five male and five female rats) administered with water by gavage; the solvent control group (C solv) (five male and five female rats) treated with 0.5% Methanol (MeOH); the positive control group (C+) (three males and three females rats) exposed to 200 mg/kg b.w. ethylmethanesulfonate (EMS). The three exposed groups (five males and five female rats per group) were treated with 7.5 + 75, 23.7 + 237, or 75 + 750 μg/kg b.w. CYN/MC-LR. All doses were prepared from a concentrate stock solution to a final volume of 1 mL with 0.5% MeOH. Although some organic solvents may affect the activity of the cytochrome P450, a 0.5% concentration of MeOH is lower than the 2% indicated to impact the activity of CYP450s [91] The number of animals included in each group was based on the OECD 474 [53] and OECD 489 [54] guidelines. Both indicate five animals of one sex per group, or five of each if both sexes are used, as an adequate number of rats. The OECD 489 [54] permits the use of a minimum of three animals of one sex, or three of each if both sexes are used, treated with a positive control. The animals for combined MN and comet assay need to be dosed at 0, 24, and 45 h, and sacrificed at 3 h after the last administration [58]. The rats were treated by gavage using an enteral feeding tube (Vygon, Ecouen, France). Clinical signs (e.g. mobility, activity, posture, blood around nose and eyes, dyspnea and piloerection) and body weight were recorded during the exposure period.

### 5.4. Sample Collection

The liver and stomach were extracted, dissected, washed with cold saline solution, and weighed. Sections of each were rapidly processed for the standard and enzyme modified comet assay as explained in Section 5.6. Furthermore, blood samples were collected and conserved in Vacutainer® sodium Heparin Tubes (Becton Dickinson, Rutherford, NJ, USA). Samples were collected from the bone marrow of both femurs of each animal for the MN assay and immediately spread on slides. The smear was then allowed to air dry, fixed with absolute methanol and stained with 10% Giemsa. Sections of liver and stomach were processed according to Diez-Quijada et al. [43] to investigate potential histopathological changes.

### 5.5. Micronucleus Assay

For this assay, two smeared glass slides (one per femur of each animal) were prepared with the bone marrow cells re-suspended in a drop of fetal bovine serum. After allowing the smear to air-dry, it was fixed in absolute methanol for five minutes and then air-dried and stained with 10% Giemsa for 10 min. The polychromatic erythrocytes (PCE)/total erythrocytes (normochromatic erythrocytes (NCE) + (PCE)) ratio and the PCE/NCE ratio were calculated by counting 500 erythrocytes per animal. The frequency of micro-nucleated immature erythrocytes (MNPCE) was expressed as % MN-PCE´s and it was determined by counting a total of 5000 PCE per animal, following Diez-Quijada et al. [43].

### 5.6. Standard and Enzyme-Modified Comet Assay 

Single cell suspensions from liver and stomach were isolated according to Corcuera et al. [92] and Diez-Quijada et al. [43] for the standard and enzyme-modified comet assay. Liver and stomach were quickly rinsed with Merchant´s buffer (MB) (0.14 M NaCl, 1.47 nM KH_2_PO_4_, 2.7 mM KCl, 8.1 mM Na_2_HPO_4_, 10 mM Na_2_EDTA, with pH 7.4). Then, a section of each tissue was homogenized in the cold by immersing it in an ice-filled beaker, and the homogenates were centrifuged, filtered, and mixed with 5 mL MB buffer before slide preparation. Heparinized blood samples were mixed with phosphate buffered saline solution (PBS) v/v (1/1), and afterwards, the lymphocytes were isolated with Histopaque^®^ (Sigma-Aldrich, Madrid, Spain) and centrifuged (30 min, 400 G). The cells were washed with PBS twice and re-suspended at a concentration of 2 × 10^5^ cells/mL in PBS.

Thirty µL of blood cell suspensions were mixed with 140 µL pre-warmed 0.5% low-melting point agarose, and 12 drops of 5 µL of each cell suspension were placed on microscope slides pre-coated with agarose. Cell suspensions of liver and stomach were mixed with 1% low-melting point agarose, and the mixtures were placed on microscope slides precoated with agarose, similar to blood samples. The standard and enzyme-modified comet assays were carried out as previously described by Diez-Quijada et al. [43]. The slides were cleaned up three times for 5 min with enzyme buffer (40 mM HEPES; 0.1 M KCl; 0.5 mM EDTA; 0.2 mg/mL bovine serum albumin; pH 8) subsequent to lysis at 4 °C. Later, two gels in each slide were exposed successively to 30 µL of each of the following: lysis solution; enzyme buffer alone (buffer F); buffer F containing Endo III; and buffer F containing Fpg in a metal box at 37 °C for 30 min. Then, the nuclei were denatured by electrophoresis carried out for 20 min, 0.81 V/cm up to 400 mA. The DNA was neutralized in PBS, washed with water and fixed with 70% and absolute ethanol. Finally, once the slides were dried, nuclei were stained with SYBR Gold and visualized with an Olympus BX61 fluorescence microscope coupled via a CCD camera to an image-analysis system (DP controller-DP manager). Images of at least 150 randomly selected nuclei per animal were analyzed with the image analysis software Comet Assay IV (Perceptive Instruments, UK). Percentages of tail DNA (% DNA in tail), automatically obtained by the software, were used to describe each of the nuclei/comets analyzed and the medians of the scored comets were obtained to describe each animal.

Endo III and Fpg sensitive sites were determined by subtracting the % of DNA in tail after repair enzymes incubation.

### 5.7. Histopathological Analysis

Tissue samples from liver and stomach were fixed in 10% phosphate-buffered formalin for 24 h, and then immediately dehydrated in ethanol, immersed in xylol, and embedded in paraffin wax employing an automatic processor. Sections of 4 µm were stained with hematoxylin and eosin and examined microscopically, with a Modular Microscopy BX43 (Olympus, Shinjuku, Tokyo, Japan). For the histopathological study, males and females were evaluated and both presented similar lesions, but the lesion score was performed only in males as they have been reported to be more sensitive to cylindrospermopsin effects [43,63]. A semiquantitative evaluation of the severity of lesions was scored. These were independently examined by two blinded and experienced observers, a veterinary pathologist and an investigator (M.A.R. and R.M.), in all the fields of one slide with a section of 1 × 1 cm from each organ studied in all the animals. The lesions scored in the liver were multinucleated hepatocytes, hepatocyte degeneration with lipid accumulation, hepatocellular necrosis, and mononuclear inflammatory periportal infiltrate, while those scored in stomach were flaking epithelium, necrosis of epithelial cells and hyperplasia of the gastric glands. Pathology scores were as follows: 0, no significant lesions (0%); 1, minimal (<10%); 2, mild (11–25%); 3, moderate (26–50%); 4, marked (51–75%); 5, severe (>75%).

### 5.8. Statistical Analysis

The results of the MN test are expressed as mean ± standard deviation (SD) for each group of animals, and a statistical analysis was carried out using the analysis of variance (ANOVA) followed by Dunnett’s multiple comparison test. The results of the standard and enzyme-modified comet assays were calculated for each group as mean ± SD of the medians. The distribution of the results was verified for normality utilizing the Kolmogorov- Smirnov test and total scores of the different groups were compared using the non-parametric Kruskal-Wallis test followed by Dunn’s multiple comparison test. Analyses were conducted using Graph-Pad InStat software (Graph-Pad Software Inc., La Jolla, San Diego, CA, USA).

## Figures and Tables

**Table 1:** Micronucleus assays results. Bone marrow cytotoxicity expressed as polychromatic erythrocytes (PCE) out of total erythrocytes (normochromatic erythrocytes (NCE) + PCE), ratio of PCE out of NCE, and the micronuclei induction expressed as % MN-PCE´s. The values are expressed as mean ± SD. Significantly different from the negative and solvent control (** *p* < 0.01).

Groups	Sex	*n*	Doses	PCE/Total	% MN-PCE´s	PCE/NCE
Negative Control (water)	♂	5		0.49 ± 0.02	0.45 ± 0.64	0.95 ± 0.08
	♀	5		0.49 ± 0.03	0.66 ± 0.32	0.96 ± 0.14
Solvent Control (0.5% MeOH)	♂	5		0.50 ± 0.02	0.83 ± 0.51	0.98 ± 0.08
	♀	5		0.50 ± 0.03	0.58 ± 0.24	1.00 ± 0.11
Positive Control (EMS *)	♂	3	200 mg/kg b.w.	0.34 ± 0.02 **	1.93 ± 0.15 **	0.51 ± 0.05 **
	♀	3		0.36 ± 0.03 **	2.47 ± 0.9 **	0.57 ± 0.08 **
CYN/MC-LR	♂	5	7.5 + 75 µg/kg b.w.	0.51 ± 0.02	1.75 ± 0.42 **	1.03 ± 0.07
	♀	5		0.50 ± 0.03	1.98 ± 0.21 **	1.05 ± 0.13
	♂	5	23.7 + 237 µg/kg b.w.	0.46 ± 0.04	1.83 ± 0.41 **	0.86 ± 0.14
	♀	5		0.47 ± 0.03	2.09 ± 0.15 **	0.88 ± 0.09
	♂	5	75 + 750 µg/kg b.w.	0.37 ± 0.03 **	1.88 ± 0.51 **	0.60 ± 0.08 **
	♀	5		0.34 ± 0.06 **	2.22 ± 0.27 **	0.51 ± 0.12 **

* EMS: ethylmethanesulfonate.

## Supplementary Materials

The following are available online at https://www.mdpi.com/2072-6651/12/6/348/s1, Table S1: Measurements (MN, alkaline comet and enzyme-modified comet assays) obtained in the target tissues (liver, stomach and blood) by dose and treatment assayed in male and female rats, Table S2: Summary statistic for treatment groups including both sexes (male and female) for the standard and enzyme-modified comet assay in liver, stomach and blood cells, Table S3: Relative weight (RW) (excised wet organ weight /animal weight) of liver and stomach from exposed male rats, Table S4: Relative weight (RW) (excised wet organ weight /animal weight) of liver and stomach from exposed female rats.

## References

[1] Glibert P.M. (2020). Harmful algae at the complex nexus of eutrophication and climate change. Harmful Algae.

[2] Buratti F.M., Manganelli M., Vichi S., Stefanelli M., Scardala S., Testai E., Funari E. (2017). Cyanotoxins: Producing organisms, occurrence, toxicity, mechanism of action and human health toxicological risk evaluation. Arch. Toxicol..

[3] Carmichael W.W. (1992). Cyanobacteria secondary metabolites – the cyanotoxins. J. Appl. Bacteriol..

[4] Environmental Protection Agency (2015). Health Effects Support Document for the Cyanobacterial Toxin Cylindrospermopsin. https://www.epa.gov/sites/production/files/2017-06/documents/cylindrospermopsin-support-report-2015.pdf.

[5] Gutiérrez-Praena D., Jos Á., Pichardo S., Moreno I.M., Cameán A.M. (2013). Presence and bioaccumulation of microcystins and cylindrospermopsin in food and the effectiveness of some cooking techniques at decreasing their concentrations: A review. Food Chem. Toxicol..

[6] Gupta N., Pant S.C., Vijayaraghavan R., Lakshmana Rao P.V. (2003). Comparative toxicity evaluation of cyanobacterial cyclic peptide toxin microcystin variants (LR, RR, YR) in mice. Toxicology.

[7] Spoof L., Catherine A. (2017). Appendix 3: Tables of microcystins and nodularins. Handb. Cyanobact. Monit. Cyanotoxin Anal..

[8] Diez-Quijada L., Prieto A.I., Guzmán-Guillen R., Jos Á., Cameán A.M. (2019). Occurrence and toxicity of microcystin congeners other than MC-LR and MC-RR: A review. Food Chem. Toxicol..

[9] Puerto M., Pichardo S., Jos Á., Cameán A.M. (2009). Comparison of the toxicity induced by microcystin-RR and microcystin-YR in differentiated and undifferentiated Caco-2 cells. Toxicon.

[10] Fischer W.J., Altheimer S., Cattori V., Meier P.J., Dietrich D.R., Hagenbuch B. (2005). Organic anion transporting polypeptides expressed in liver and brain mediate uptake of microcystin. Toxicol. Appl. Pharmacol..

[11] MacKintosh C., Beattie K.A., Klumpp S., Cohen P., Codd G.A. (1990). Cyanobacterial microcystin-LR is a potent and specific inhibitor of protein phosphatases 1 and 2A from both mammals and higher plants. FEBS Lett..

[12] Falconer I.R., Yeung D.S.K. (1992). Cytoskeletal changes in hepatocytes induced by Microcystis toxins and their relation to hyperphosphorylation of cell proteins. Chem. Biol. Interact..

[13] Chorus I., Bartram J., Chorus I., Bertram J. (1999). Toxic Cyanobacteria in Water: A Guide to Their Public Health Consequences, Monitoring and Management.

[14] Guzman R.E., Solter P.F. (1999). Hepatic oxidative stress following prolonged subletal Microcystin-LR exposure. Toxicol. Pathol..

[15] Ding W.-X., Shen H.-M., Ong C.-N. (2001). Critical role of reactive oxygen species formation in microcystin-induced cytoskeleton disruption in primary cultured hepatocytes. J. Toxicol. Environ. Health A.

[16] Žegura B., Lah T.T., Filipič M. (2004). The role of reactive oxygen species in microcystin-LR induced DNA damage. Toxicology.

[17] Puerto M., Pichardo S., Jos Á., Prieto A.I., Sevilla E., Frías J.E., Cameán A.M. (2010). Differential oxidative stress response to pure Microcystin-LR and Microcystin-containing and non-containing cyanobacterial crude extracts on Caco-2 cells. Toxicon.

[18] Nishiwaki-Matsushima R., Ohta T., Nishiwaki S., Suganuma M., Kohyama K., Ishikawa T., Carmichael W.W., Fujiki H. (1992). Liver tumor promotion by the cyanobacterial cyclic peptide toxin microcystin-LR. J. Cancer Res. Clin. Oncol..

[19] Abramsson-Zetterberg L., Sundh U.B., Mattsson R. (2010). Cyanobacterial extracts and microcystin-LR are inactive in the micronucleus assay in vivo and in vitro. Mutat. Res..

[20] Žegura B. (2016). An overview of the mechanisms of microcystin-LR genotoxicity and potential carcinogenicity. Mini Rev. Med. Chem..

[21] IARC Working Group on the Evaluation of Carcinogenic Risks to Humans (2010). Ingested Nitrate and Nitrite, and Cyanobacterial Peptide Toxins. https://www.ncbi.nlm.nih.gov/books/NBK326544/pdf/Bookshelf_NBK326544.pdf.

[22] Žegura B., Štraser A., Filipič M. (2011). Genotoxicity and potential carcinogenicity of cyanobacterial toxins – a review. Mutat. Res.-Rev. Mutat. Res..

[23] Rao P.V.L., Bhattacharya R. (1996). The cyanobacterial toxin Microcystin-LR induced DNA damage in mouse liver in vivo. Toxicology.

[24] Rao P.V.L., Gupta N., Jayaraj R., Bhaskar A.S.B., Jatav P.C. (2005). Age-dependent effects on biochemical variables and toxicity induced by cyclic peptide toxin microcystin-LR in mice. Comp. Biochem. Physiol. C Toxicol. Pharmacol..

[25] Gaudin J., Huet S., Jarry G., Fessard V. (2008). in vivo DNA damage induced by the cyanotoxin microcystin-LR: Comparison of intra-peritoneal and oral administrations by use of the comet assay. Mutat. Res..

[26] Dias E., Louro H., Pinto M., Santos T., Antunes S., Pereira P., Silva M.J. (2014). Genotoxicity of microcystin-LR in in vitro and in vivo experimental models. BioMed. Res. Int..

[27] Zhou W., Zhang X., Xie P., Liang H., Zhang X. (2013). The suppression of hematopoiesis function in Balb/c mice induced by prolonged exposure of microcystin-LR. Toxicol. Lett..

[28] Gaudin J., Le Hegarat L., Nesslany F., Marzin D., Fessard V. (2009). In vivo genotoxic potential of microcystin-LR: A cyanobacterial toxin, investigated both by the unscheduled DNA synthesis (UDS) and the comet assays after intravenous administration. Environ. Toxicol..

[29] Zhan L., Honma M., Wang L., Hayashi M., Wu D.-S., Zhang L.-S., Rajaguru P., Suzuki T. (2006). Microcystin-LR is not Mutagenic in vivo in the *λ*/*lacZ* Transgenic Mouse (Muta^TM^Mouse). Gene. Environ..

[30] Ohtani I., Moore R.E., Runnegar M.T.C. (1992). Cylindrospermopsin: A potent hepatotoxin from the blue-green alga *Cylindrospermopsis raciborskii*. J. Am. Chem. Soc..

[31] Kinnear S. (2010). Cylindrospermopsin: A decade of progress on bioaccumulation research. Mar. Drugs.

[32] Manning S.R., Nobles D.R. (2017). Impact of global warming on water toxicity: Cyanotoxins. Curr. Opin. Food Sci..

[33] Pichardo S., Cameán A.M., Jos Á. (2017). In vitro toxicological assessment of Cylindrospermopsin: A review. Toxins.

[34] Terao K., Ohmori S., Igarashi K., Ohtani I., Watanabe M.F., Harada K.I., Ito E., Watanabe M. (1994). Electron microscopic studies on experimental poisoning in mice induced by cylindrospermopsin isolated from blue-green alga *Umezakia natans*. Toxicon.

[35] Runnegar M.T., Kong S.-M., Zhong Y.-Z., Lu S.C. (1995). Inhibition of reduced glutathione synthesis by cyanobacterial alkaloid cylindrospermopsin in cultured rat hepatocytes. Biochem. Pharmacol..

[36] Froscio S.M., Humpage A.R., Burcham P.C., Falconer I.R. (2003). Cylindrospermopsin-induced protein synthesis inhibition and its dissociation from acute toxicity in mouse hepatocytes. Environ. Toxicol. Int. J..

[37] Puerto M., Jos Á., Pichardo S., Moyano R., Blanco A., Cameán A.M. (2014). Acute exposure to pure Cylindrospermopsin results in oxidative stress and pathological alterations in Tilapia (*Oreochromis niloticus*). Environ. Toxicol..

[38] Humpage A.R., Fontaine F., Froscio S., Burcham P., Falconer I.R. (2005). Cylindrospermopsin genotoxicity and cytotoxicity: Role of cytochrome P-450 and oxidative stress. J. Toxicol. Environ. Health A.

[39] Bazin E., Mourot A., Humpage A.R., Fessard V. (2010). Genototoxicity of a freshwater cyanotoxin, cylindrospermopsin, in two human cell lines: Caco-2 and HepaRG. Environ. Mol. Mutat..

[40] Shen X., Lam P.K.S., Shaw G.R., Wickramasinghe W. (2002). Genotoxity investigation of a cyanobacterial toxin, cylindrospermopsin. Toxicon.

[41] Bazin E., Huet S., Jarry G., Le Hégarat L., Munday J.S., Humpage A.R., Fessard V. (2012). Cytotoxic and genotoxic effects of cylindrospermopsin in mice treated by gavage or intraperitoneal injection. Environ. Toxicol..

[42] Dordevic N.B., Matíc S.L.J., Simić S.B., Stanić S.M., Mihailivić V.B., Stancović N.M., Stancović V.D., Cirić A.R. (2017). Impact of the toxicity of *Cylindrospermopsis raciboskii* (Woloszynska) Seenayya & Subba Raju on laboratory rats in vivo. Environ. Sci. Pollut. Res..

[43] Diez-Quijada L., Llana-Ruiz-Cabello M., Cătunescu M.G., Puerto M., Moyano R., Jos Á., Cameán A.M. (2019). In vivo genotoxicity evaluation of cylindrospermopsin in rats using a combined micronucleus and comet assay. Food. Chem. Toxicol..

[44] Bittencourt-Oliveira M., Carmo D., Piccin-Santos V., Moura A.N., Aragão-Tavares N.K., Cordeiro-Araújo M.K. (2014). Cyanobacteria, microcystins and cylindrospermopsin in public drinking supply reservoirs of Brazil. An. Acad. Bras. Cienc..

[45] Jančula D., Straková L., Sadílek J., Maršálek B., Babica P. (2014). Survey of cyanobacterial toxins in Czech water reservoirs—The first observation of neurotoxic saxitoxins. Environ. Sci. Pollut. Res. Int..

[46] León C., Peñuela G.A. (2019). Detected cyanotoxins by UHPLC MS/MS technique in tropical reservoirs of northeastern Colombia. Toxicon.

[47] Testai E., Buratti F.M., Funari E., Manganelli M., Vichi S., Arnich N., Biré R., Fessard V., Sialehaamoa A. (2016). Review and analysis of occurrence, exposure and toxicity of cyanobacteria toxins in food. EFSA Support. Publ..

[48] Hercog K., Maisanaba S., Filipič M., Jos Á., Cameán A.M., Žegura B. (2017). Genotoxic potential of the binary mixture of cyanotoxins microcystin-LR and cylindrospermopsin. Chemosphere.

[49] Diez-Quijada L., Prieto A.I., Jos Á., Cameán A.M. (2019). in vitro mutagenic and genotoxic assessment of a mixture of the cyanotoxins Microcystin-LR and Cylindrospermopsin. Toxins.

[50] Gutiérrez-Praena D., Guzmán-Guillén R., Pichardo S., Moreno F.J., Vasconcelos V., Jos Á., Cameán A.M. (2018). Cytotoxic and morphological effects of microcystin-LR, cylindrospermopsin, and their combinations on the human hepatic cell line HepG2. Environ. Toxicol..

[51] Hinojosa M.G., Prieto A.I., Gutiérrez-Praena D., Moreno F.J., Cameán A.M., Jos Á. (2019). Neurotoxic assessment of Microcystin-LR, cylindrospermopsin and their combination on the human neuroblastoma SH-SY5Y cell line. Chemosphere.

[52] EFSA Scientific Committee (2011). Scientific opinion on genotoxicity testing strategies applicable to food and feed safety assessment. EFSA J..

[53] (2016). OECD Guidelines for the Testing of Chemicals: Mammalian Erythrocyte Micronucleus Test. https://www.oecd.org/env/test-no-474-mammalian-erythrocyte-micronucleus-test-9789264264762-en.htm.

[54] (2016). OECD Guideline for the Testing of Chemicals: In vivo Mammalian Alkaline Comet Assay. https://www.oecd.org/env/test-no-489-in-vivo-mammalian-alkaline-comet-assay-9789264264885-en.htm.

[55] Zervou S.K., Christophoridis C., Kaloudis T., Triantis T.M., Hiskia A. (2017). New SPE-LC-MS/MS method for simultaneous determination of multi-class cyanobacterial and algal toxins. J. Hazard. Mater..

[56] Pinheiro C., Azevedo J., Campos A., Vasconcelos V., Loureiro S. (2016). The interactive effects of microcystin-LR and cylindrospermopsin on the growth rate of the freshwater algae *Chlorella vulgaris*. Ecotoxicology.

[57] Puerto M., Prieto A.I., Maisanaba S., Gutiérrez-Praena D., Mellado-García P., Jos Á., Cameán A.M. (2018). Mutagenic and genotoxic potential of pure Cylindrospermopsin by a battery of in vitro tests. Food Chem. Toxicol..

[58] Bowen D.E., Whitwell J.H., Lillford L., Henderson D., Kidd D., McGarry S., Pearce G., Beevers C., Kirkland D.J. (2011). Evaluation of a multi-endpoint assay in rats, combining the bone-marrow micronucleus test, the comet assay and the flow-cytometric peripheral blood micronucleus test. Mutat. Res. Genet. Toxicol. Environ. Mutagen.

[59] Kirkland D., Levy D.D., LeBaron M.J., Aardema M.J., Beevers C., Bhalli J., Douglas G.R., Escobar P.A., Farabaugh C.S., Guerard M. (2019). A comparison of transgenic rodent mutation and in vivo comet assay responses for 91 chemicals. Mutat. Res. Genet.Toxicol. Environ. Mutagen..

[60] Azqueta A., Shaposhnikov S., Collins A.R. (2009). DNA oxidation: Investigating its key role in environmental mutagenesis with the comet assay. Mutat. Res. Genet. Toxicol. Environ. Mutagen..

[61] Llana-Ruiz-Cabello M., Maisanaba S., Puerto M., Prieto A.I., Pichardo S., Moyano R., González-Pérez J.A., Cameán A.M. (2016). Genotoxicity evaluation of carvacrol in rats using a combined micronucleus and comet assay. Food Chem. Toxicol..

[62] Hooser S.B., Beasley V.R., Lovell R.A., Carmichael W.W., Haschek W.M. (1989). Toxicity of Microcystin LR, a Cyclic Heptapeptide Hepatotoxin from *Microcystis aeruginosa*, to Rats and Mice. Vet. Pathol..

[63] Chernoff N., Hill D.J., Chorus I., Diggs D.L., Huang H., King D., Lang J.R., Le T.T., Schmid J.E., Travlos G.S. (2018). Cylindrospermopsin toxicity in mice following a 90-d oral exposure. J. Toxicol. Environ. Health A.

[64] Diez-Quijada L., Puerto M., Gutiérrez-Praena D., Llana-Ruiz-Cabello M., Jos Á., Cameán A.M. (2019). Microcystin-RR: Occurrence, content in water and food and toxicological studies. A review. Environ. Res..

[65] Humpage A.R., Fenech M., Thomas P., Falconer I.R. (2000). Micronucleus induction and chromosome loss in transformed human white cells indicate clastogenic and aneugenic action of the cyanobacterial toxin, cylindrospermopsin. Mutat. Res. Genet. Toxicol. Environ. Mutagen..

[66] Sieroslawska A., Rymuszka A. (2015). Cylindrospermopsin induces oxidative stress and genotoxic effects in the fish CLC cell line. J. Appl. Toxicol..

[67] Štraser A., Žegura B., Filipič M. (2011). Genotoxic effects of the cyanobacterial hepatotoxin cylindrospermopsin in the HepG2 cell line. Arch. Toxicol..

[68] Zhan L., Sakamoto H., Sakuraba M., Wu D.-S., Zhang L.-S., Suzuki T., Hayashi M., Honma M. (2004). Genotoxicity of microcystin-LR in human lymphoblastoid TK6 cells. Mutat. Res. Genet. Toxicol. Environ. Mutagen..

[69] Štraser A., Filipič M., Gorenc I., Žegura B. (2013). The influence of cylindrospermopsin on oxidative DNA damage and apoptosis induction in HepG2 cells. Chemosphere.

[70] Žegura B., Sedmak B., Filipič M. (2003). Microcystin-LR induces oxidative DNA damage in human hepatoma cell line HepG2. Toxicon.

[71] Žegura B., Gajski G., Štraser A., Garaj-Vrhovac V., Filipič M. (2011). Microcystin-LR induced DNA damage in human peripheral blood lymphocytes. Mutat. Res. Genet. Toxicol. Environ. Mutagen..

[72] Amé M.V., Baroni M.V., Galanti L.N., Bocco J.L., Wunderlin D.A. (2009). Effects of microcystin-LR on the expression of P-glycoprotein in *Jenynsia multidentata*. Chemosphere.

[73] Qiu T., Xie P., Liu Y., Li G., Xiong Q., Hao L., Li H. (2009). The profound effects of microcystin on cardiac antioxidant enzymes, mitochondrial function and cardiac toxicity in rat. Toxicology.

[74] Arman T., Lynch K.D., Montonye M.L., Goedken M., Clarke J.D. (2019). Sub-Chronic Microcystin-LR Liver Toxicity in Preexisting Diet-Induced Nonalcoholic Steatohepatitis in Rats. Toxins.

[75] Yoshida T., Makita Y., Nagata S., Tsutsumi T., Yoshida F., Sekijima M., Tamura S.I., Ueno Y. (1997). Acute oral toxicity of microcystin-LR, a cyanobacterial hepatotoxin, in mice. Nat Toxins.

[76] Sedan D., Andrinolo D., Telese L., Giannuzzi L., de Alaniz M.J., Marra C.A. (2010). Alteration and recovery of the antioxidant system induced by sub-chronic exposure to microcystin-LR in mice: Its relation to liver lipid composition. Toxicon.

[77] Zhang X.X., Zhang Z., Fu Z., Wang T., Qin W., Xu L., Cheng S., Yang L. (2010). Stimulation effect of microcystin-LR on matrix metalloproteinase-2/-9 expression in mouse liver. Toxicol. Lett..

[78] Sun X., Mi L., Liu J., Song L., Chung F.L., Gan N. (2011). Sulforaphane prevents microcystin-LR-induced oxidative damage and apoptosis in BALB/c mice. Toxicol. Appl. Pharmacol..

[79] Weng D., Lu Y., Wei Y., Liu Y., Shen P. (2007). The role of ROS in microcystin-LR-induced hepatocyte apoptosis and liver injury in mice. Toxicology.

[80] Han Z.X., Yang L., Zhang L., Xu C., Shu W.Q. (2010). The antagonistic action of epigallocatechin-3-gallate on microcystin LR-induced oxidative damage on hepatocytes of mice and the expression of cytochrome P450 2E1. Zhonghua Yu Fang Yi Xue Za Zhi.

[81] Sibaldo de Almeida C., Costa de Arruda A.C., Caldas de Queiroz E., Matias de Lima Costa H.T., Fernandes Barbosa P., Araújo Moura Lemos T.M., Nunes Oliveira C., Pinto E., Schwarz A., Kujbida P. (2013). Oral exposure to cylindrospermopsin in pregnant rats: Reproduction and foetal toxicity studies. Toxicon.

[82] Humpage A.R., Falconer I.R. (2003). Oral toxicity of the cyanobacterial toxin cylindrospermopsin in male Swiss albino mice: Determination of no observed adverse effect level for deriving a drinking water guideline value. Environ. Toxicol..

[83] Chernoff N., Rogers E.H., Zehr R.D., Gage M.I., Malarkey D.E., Bradfield C.A., Liu Y., Schmid J.E., Jaskot R.H., Richards J.H. (2010). Toxicity and recovery in the pregnant mouse after gestational exposure to the cyanobacterial toxin, cylindrospermopsin. J. Appl. Toxicol..

[84] Seawright A.A., Nolan C.C., Shaw G.R., Chiswell R.K., Norris R.L., Moore M.R., Smith M.J. (1999). The oral toxicity for mice of the tropical cyanobacterium *Cylindrospermopsis raciborskii* (Wolonszynska). Environ. Toxicol..

[85] Pouria S., de Andrade A., Barbosa J., Cavalcanti R.L., Barreto V.T., Ward C.J., Preiser W., Poon G.K., Neild G.H., Codd G.A. (1998). Fatal microcystin intoxication in haemodialysis unit in Caruaru, Brazil. Lancet.

[86] Frangež R., Kosec M., Sedmak B., Beravs K., Demsar F., Juntes P., Pogačnik M., Šuput D. (2000). Subchronic liver injuries caused by microcystins. Pflügers Arch..

[87] Ito E., Kondo F., Harada K.I. (1997). Hepatic necrosis in aged mice by oral administration of microcystin-LR. Toxicon.

[88] Iwabuchi T., Iijima K., Ara N., Koike T., Shinkai H., Ichikawa T., Kamata Y., Ishihara K., Shimosegawa T. (2013). Increased gastric mucus secretion alleviates non-steroidal anti-inflammatory drug-induced abdominal pain. Tohoku J. Exp. Med..

[89] De La Cruz A.A., Hiskia A., Kaloudis T., Chernoff N., Hill D., Antoniou M.G., He X., Loftin K., O’Shea K., Zhao C. (2013). A review on cylindrospermopsin: The global occurrence, detection, toxicity and degradation of a potent cyanotoxin. Environ. Sci. Process. Impacts.

[90] International Conference of Harmonisation (ICH) (2012). Guidance on Genotoxicity Testing and Data Interpretation for Pharmaceuticals Intended for Human Use S2 (R1). https://www.fda.gov/regulatory-information/search-fda-guidance-documents/s2r1-genotoxicity-testing-and-data-interpretation-pharmaceuticals-intended-human-use.

[91] Easterbrook J., Lu C., Sakai Y., Li A.P. (2001). Effects of organic solvents on the activities of cytochrome P450 isoforms, UDP-dependent glucuronyl transferase, and phenol sulfotransferase in human hepatocytes. Drug Metab. Dispos..

[92] Corcuera L.A., Vettorazzi A., Arbillaga L., Pérez N., Gil A.G., Azqueta A., González-Peñas E., García-Jalón J.A., López de Cerain A. (2015). Genotoxicity of Aflatoxin B1 and Ochratoxin A after simultaneous application of the in vivo micronucleus and comet assay. Food Chem. Toxicol..

