# Supplementary material for: Cylindrospermopsin-Microcystin-LR Combinations May Induce Genotoxic and Histopathological Damage in Rats
Source: Toxins (Basel). 2020 May 26;12(6):348. doi: 10.3390/toxins12060348 (PMC7354441; doi:10.3390/toxins12060348)
Supplement: Supplementary file 1 [file toxins-12-00348-s001.pdf]

Supplementary Materials: Cylindrospermopsin-Microcystin-LR Combinations May Induce Genotoxic and Histopathological Damage in Rats

Leticia Díez-Quijada, Concepción Medrano-Padial, María Llana-Ruiz-Cabello, Giorgiana M. Cătunescu, Rosario Moyano, Maria A. Risalde, Ana M. Cameán and Ángeles Jos

Table S1. Measurements (MN, alkaline comet and enzyme-modified comet assays) obtained in the target tissues (liver, stomach and blood) by dose and treatment assayed in male and female rats.

**Doses**	**Genotoxicity Measurements (Males)**									
					**Enzyme-Modified Comet Assay**					
	**% MN Test**	**Standard Comet Assay**			**Endo III-Sensitive Sites**			**Fpg-Sensitive Sites**		
	**Bone Marrow**	**Liver**	**Stomach**	**Blood**	**Liver**	**Stomach**	**Blood**	**Liver**	**Stomach**	**Blood**
Negative control (water)	1.6	0.60	3.45	0.15	8.9	1.28	0.15	2.70	2.50	0.75
	0.22	0.00	0.01	0.25	1.2	9.43	4.22	10.10	12.00	0.35
	0.16	0.00	0.01	0.22	3.1	10.0	0.70	4.62	2.95	0.08
	0.16	0.05	4.50	0.12	10.0	8.4	1.10	10.60	4.50	0.05
	0.12	0.68	0.00	0.18	6.40	8.8	0.00	3.50	5.80	0.10
Solvent control (0.5% MeOH)	1.58	0.50	6.02	0.00	3.34	1.03	2.3	2.90	4.31	0.04
	1.10	0.00	4.00	3.20	0.44	0.16	0.03	2.95	1.15	0.45
	0.34	0.00	0.00	0.00	0.51	0.30	0.03	2.90	0.01	0.01
	0.48	0.20	0.00	0.05	0.74	0.53	0.70	8.00	0.00	0.10
	0.64	0.50	5.80	0.07	11.60	5.64	0.01	7.30	0.00	0.06
Positive Control	1.84	34.34	43.48	37.28	29.05	40.2	27.4	25.70	40.7	33.60
	2.10	16.52	17.42	15.70	25.35	32.9	30.4	23.15	35.0	25.80
	1.84	22.89	35.11	40.91	19.3	37.2	24.4	31.95	33.45	28.80
7.5 + 75 µg/kg b.w. CYN/MC-LR	2.40	0.50	1.50	0.01	12.9	12.6	0.41	9.18	8.30	0.07
	1.54	0.25	2.30	0.25	7.55	16.4	0.00	6.34	14.08	0.07
	1.95	0.15	2.10	0.40	7.19	9.85	1.99	5.19	6.35	0.08
	1.50	0.16	0.90	0.05	8.18	10.45	0.20	8.66	13.34	0.26
	1.36	0.80	1.50	0.22	9.34	12.54	0.42	14.55	14.68	0.49
23.7 + 237 µg/kg b.w. CYN/MC-LR	2.34	0.23	0.09	2.15	0.63	6.10	0.09	0.80	3.34	0.01
	1.28	0.33	0.06	0.1	3.11	0.35	0.06	0.60	2.95	0.01
	1.68	0.20	0.02	0.09	0.08	4.65	0.40	0.20	3.26	0.05
	2.10	0.27	0.05	0.04	0.76	8.80	0.02	0.35	10.98	0.02
	1.74	0.12	0.10	0.00	0.42	5.10	0.07	0.60	11.87	0.02
75 + 750 µg/kg b.w. CYN/MC-LR	2.68	2.18	9.70	0.65	9.0	6.20	0.00	15.45	12.95	0.07
	1.54	2.00	1.02	0.10	6.9	4.80	0.04	6.85	7.00	0.20
	1.48	1.23	0.50	0.00	4.7	4.20	0.04	8.70	4.70	0.13
	1.60	1.17	0.45	0.05	2.6	5.30	0.09	3.50	8.00	0.07
	2.12	1.82	9.20	6.90	1.7	4.75	0.58	3.75	5.70	3.64

**Doses**	**Genotoxicity Measurements (Females)**									
					**Enzyme-Modified comet assay**					
	**% MN Test**	**Standard Comet Assay**			**Endo III-Sensitive Sites**			**Fpg-Sensitive Sites**		

	**Bone Marrow**	**Liver**	**Stomach**	**Blood**	**Liver**	**Stomach**	**Blood**	**Liver**	**Stomach**	**Blood**
Negative control (water)	0.84	0.59	0.00	0.75	0.17	11.4	0.64	1.85	6.60	2.59
	1.14	1.80	0.20	0.82	0.30	4.1	3.59	1.20	8.40	0.49
	0.48	0.17	0.45	0.08	0.82	12.7	0.00	3.85	12.90	0.01
	0.44	0.03	0.50	1.17	3.79	10.7	0.04	3.80	5.40	0.01
	0.38	0.17	1.30	0.03	2.76	11.9	1.10	5.30	10.4	1.20
Solvent control (0.5% MeOH)	0.70	1.43	0.07	0.02	0.60	5.79	1.10	2.16	7.36	1.60
	0.20	1.48	0.07	0.04	0.63	2.54	0.03	3.57	4.52	1.60
	0.84	0.28	0.05	0.05	0.46	3.06	2.05	1.56	11.22	2.15
	0.54	0.44	0.06	0.04	2.03	6.19	0.35	2.27	7.83	0.01
	0.60	1.08	0.10	0.03	2.03	8.22	4.50	4.98	4.86	6.10
Positive Control	3.28	23.80	37.50	29.35	23.2	46.95	30.0	24.01	37.90	31.30
	1.50	19.73	37.90	12.54	28.8	43.6	24.05	21.88	40.40	26.95
	2.64	23.44	39.00	36.22	29.7	49.7	29.2	27.57	35.50	25.00
7.5 + 75 µg/kg b.w. CYN/MC-LR	2.04	0.10	4.90	0.14	1.45	9.70	0.2	1.75	7.40	0.95
	2.30	0.93	5.90	0.16	7.38	14.40	3.9	8.70	10.20	0.08
	1.92	0.62	1.95	0.16	0.70	7.00	2.6	1.70	8.30	0.40
	1.92	0.03	2.10	0.03	1.80	11.5	2.7	2.60	10.50	2.80
	1.72	0.99	4.45	0.03	2.64	11.00	1.02	4.90	3.60	5.10
23.7 + 237 µg/kg b.w. CYN/MC-LR	2.02	0.48	1.90	0.70	0.41	6.90	0.02	0.00	2.00	0.02
	2.26	0.09	2.70	0.34	0.99	5.60	1.29	0.35	1.05	0.07
	2.12	2.71	2.21	0.06	2.05	8.90	0.07	0.40	7.40	0.00
	1.86	0.12	2.15	0.01	1.73	2.90	0.02	1.50	6.50	0.00
	2.18	0.11	2.95	0.53	1.49	3.70	0.01	4.00	3.60	0.00
75 + 750 µg/kg b.w. CYN/MC-LR	2.08	1.20	1.00	0.10	4.93	3.62	3.86	4.10	8.00	2.47
	2.34	0.05	1.15	1.76	0.43	1.95	0.44	1.00	5.00	0.57
	2.40	0.10	2.95	0.03	0.15	7.02	0.28	1.35	6.60	0.94
	1.80	0.95	1.60	1.38	1.93	1.93	0.64	0.05	11.40	0.30
	2.46	1.20	1.64	2.00	0.02	7.00	0.77	1.00	10.50	0.22

The values are expressed as individual measurements.

Table S2. Summary statistic for treatment groups including both sexes (male and female) for the standard and enzyme-modified comet assay in liver, stomach and blood cells.

				**Enzyme-Modified Comet Assay**					
	**Standard Comet Assay**			**Endo-III Sensitives Sites**			**Fpg-Sensitives Sites**		
	**Liver (mean ± SD)**	**Stomach (mean ± SD)**	**Blood** (mean ± SD)	**Liver** (mean ± SD)	**Stomach** (mean ± SD)	**Blood** (mean ± SD)	**Liver** (mean ± SD)	**Stomach** (mean ± SD)	**Blood** (mean ± SD)
Negative control (water)	0.41 ± 0.56	1.04 ± 1.62	0.38 ± 0.39	3.74 ± 3.56	8.87 ± 3.59	1.15 ± 1.52	4.75 ± 3.19	7.15 ± 3.65	0.56 ± 0.81

Solvent control (0.5% MeOH)	0.60 ± 0.58 (1.47-fold)	1.62 ± 2.58 (1.55-fold)	0.38 ± 1.06 (1.01-fold)	2.24 ± 3.43 (0.59-fold)	3.35 ± 2.92 (0.38-fold)	0.84 ± 1.21 (0.73-fold)	3.86 ± 2.20 (0.81-fold)	4.13 ± 3.87 (0.58-fold)	1.21 ± 1.90 (2.15-fold)
7.5 + 75 μg/kg CYN/MC-LR	0.45 ± 0.36 (1.11-fold)	2.76 ± 1.69 (2.65-fold)	0.15 ± 0.12 (0.38-fold)	5.91 ± 4.04 (1.58-fold)	11.54 ± 2.63 (1.30-fold)	1.34 ± 1.36 (1.16-fold)	6.36 ± 4.03 (1.34-fold)	9.68 ± 3.59 (1.35-fold)	1.03 ± 1.65 (1.83-fold)
23.7 + 237 μg/kg CYN/MC-LR	0.47 ± 0.80 (1.14-fold)	1.22 ± 1.26 (1.17-fold)	0.40±0.66 (1.07-fold)	1.17 ± 0.93 (0.31-fold)	5.3 ± 2.62 (0.60-fold)	0.21 ± 0.40 (0.18-fold)	0.88 ± 1.17 (0.19-fold)	5.30 ± 3.75 (0.74-fold)	0.02 ± 0.02 (0.04-fold)
75 + 750 μg/kg CYN/MC-LR	1.19 ± 0.71 (2.91-fold)	2.92 ± 3.51 (2.80-fold)	1.30±2.11 (3.44-fold)	3.24 ± 3.05 (0.86-fold)	4.68 ± 1.82 (0.53-fold)	0.67 ± 1.15 (0.58-fold)	4.58 ± 4.70 (0.96-fold)	7.99 ± 2.80 (1.12-fold)	0.86 ± 1.22 (1.53-fold)
Positive control (EMS)	23.45 ± 6.01*** (57.34-fold)	35.07 ± 9.07*** (33.65-fold)	28.67 ± 11.91*** (76.04-fold)	25.9 ± 4.10** (6.92-fold)	41.76 ± 6.25** (4.71-fold)	27.58 ± 2.79** (23.90-fold)	25.71 ± 3.65** (5.41-fold)	37.16 ± 2.99** (5.2-fold)	28.58 ± 3.34** (50.75-fold)

Negative control: Water; Solvent control: 0.5% MeOH; CYN: Cylindrospermopsin; MC-LR: Microcystin-LR; Positive control : Ethylmethanesulfonate (EMS). The levels of DNA strand breaks are expressed as % DNA in the tail of the comets. All values are represented as mean ± SD. Significantly different from control (**p<0.01; ***p < 0.001).

Table S3. Relative weight (RW) (excised wet organ weight /animal weight) of liver and stomach from exposed male rats.

	Male					
	Negative control (Water) n = 5 (mean ± SD)	Solvent Control (0.5% MeOH) n = 5 (mean ± SD)	7.5 + 75 μg/mL CYN/MC-LR n = 5 (mean ± SD)	23.7 + 237 μg/mL CYN/MC-LR n = 5 (mean ± SD)	75+750 μg/mL CYN/MC-LR n = 5 (mean ± SD)	Positive control (EMS) n =3 (mean ± SD)
Body weight (g)	145.19 ± 27.56	156.54 ± 24.26	132.53 ± 12.18	164.75 ± 5.42	146.88 ± 15.75	138.38 ± 14.86
Liver	3.14 ± 0.03	3.22 ± 0.17	3.25 ± 0.11	3.02 ± 0.17	3.24 ± 0.25	3.52 ± 0.24
Stomach	0.49 ± 0.06	0.38 ± 0.05	0.52 ± 0.09	0.47 ± 0.09	0.46 ± 0.15	0.84 ± 0.30**

Negative control: water; Solvent control: 0.5% MeOH; CYN: Cylindrospermopsin; MC-LR: Microcystin-LR; Positive control : Ethylmethanesulfonate (EMS). The values are expressed as mean ± SD. Significantly different from the negative control (**p < 0.01).

Table S4. Relative weight (RW) (excised wet organ weight /animal weight) of liver and stomach from exposed female rats.

	Female					
	Negative Control (Water) n = 5 (mean ± SD)	Solvent Control (0.5% MeOH) n = 5 (mean ± SD)	7.5 + 75 μg/mL CYN/MC-LR n = 5 (mean ± SD)	23.7 + 237 μg/mL CYN/MC-LR n = 5 (mean ± SD)	75 + 750 μg/mL CYN/MC-LR n = 5 (mean ± SD)	Positive Control (EMS) n =3 (mean ± SD)
Body weight (g)	168.35 ± 15.57	173.53 ± 16.29	175.20 ± 7.28	165.46 ± 12.05	171.60 ± 9.44	172.30 ± 17.16
Liver	3.07 ± 0.17	2.97 ± 0.22	2.90 ± 0.19	2.96 ± 0.12	3.09 ± 0.16	3.56 ± 0.41*

Stomach	0.39 ± 0.06	0.38 ± 0.06	0.33 ± 0.02	0.43 ± 0.09	0.52 ± 0.14	0.53 ± 0.26

Negative control: Water; Solvent control: 0.5% MeOH; CYN: Cylindrospermopsin; MC-LR: Microcystin-LR; Positive control +: Ethylmethanesulfonate (EMS). The values are expressed as mean ± SD. Significantly different from the negative control (*$p < 0.05$)
